# Molecular breeding of pigs in the genome editing era

**DOI:** 10.1186/s12711-025-00961-7

**Published:** 2025-03-10

**Authors:** Jiahuan Chen, Jiaqi Wang, Haoran Zhao, Xiao Tan, Shihan Yan, Huanyu Zhang, Tiefeng Wang, Xiaochun Tang

**Affiliations:** 1 College of Animal Sciences, Jilin University, Changchun, 130062 China; 2 College of Life Science, Baicheng Normal University, Baicheng, 137000 China

## Abstract

**Background:**

To address the increasing demand for high-quality pork protein, it is essential to implement strategies that enhance diets and produce pigs with excellent production traits. Selective breeding and crossbreeding are the primary methods used for genetic improvement in modern agriculture. However, these methods face challenges due to long breeding cycles and the necessity for beneficial genetic variation associated with high-quality traits within the population. This limitation restricts the transfer of desirable alleles across different genera and species. This article systematically reviews past and current research advancements in porcine molecular breeding. It discusses the screening of clustered regularly interspaced short palindromic repeats (CRISPR) to identify resistance loci in swine and the challenges and future applications of genetically modified pigs.

**Main body:**

The emergence of transgenic and gene editing technologies has prompted researchers to apply these methods to pig breeding. These advancements allow for alterations in the pig genome through various techniques, ranging from random integration into the genome to site-specific insertion and from target gene knockout (KO) to precise base and prime editing. As a result, numerous desirable traits, such as disease resistance, high meat yield, improved feed efficiency, reduced fat deposition, and lower environmental waste, can be achieved easily and effectively by genetic modification. These traits can serve as valuable resources to enhance swine breeding programmes.

**Conclusion:**

In the era of genome editing, molecular breeding of pigs is critical to the future of agriculture. Long-term and multidomain analyses of genetically modified pigs by researchers, related policy development by regulatory agencies, and public awareness and acceptance of their safety are the keys to realizing the transition of genetically modified products from the laboratory to the market.

## Background

Pigs play a vital role as both agricultural and economic animals, serving as a primary source of meat in many countries. The history of pig breeding reflects a global exchange of genetics. Initially focussed on selecting for appearance, breeding practices evolved to prioritise fat pigs but now there is demand for leaner varieties. The demand for different pig breeds has adapted over time to align with the economic, social, and environmental conditions of each era. Traditional crossbreeding and selective breeding have successfully improved the quality and efficiency of pig production. In the United States, reports indicate that, from 1959 to 2009, the feed-to-gain ratio has been reduced by 33% and the carbon footprint by 35% [1]. However, the swine industry still faces many challenges, including the impact of various infectious viruses [2, 3], the tendency for dietary energy to contribute to the accumulation of adipose tissue [4], and the inadequate digestion of plant-based proteins, minerals, and fibres. Traditional breeding programs typically require a lengthy selection period, and most importantly, they can only achieve genetic improvements when beneficial natural genetic variations are present in the population. If there is no genetic variation, there is no possibility of improving traits by selective breeding.

The emergence and innovation of modern breeding biotechniques, ranging from transgenic techniques to gene editing strategies, have introduced new options for developing high-quality pig breeds. These advances have made it possible to shift from traditional breeding to modern molecular breeding that focuses on gene manipulation.

Researchers initially employed transgenic technology to achieve stable expression of exogenous genes in pigs, which helped overcome reproductive isolation between species. However, these previous methods of gene integration have proven to be inefficient, and the exact locations where integration occurs are often uncertain. The production of beneficial traits does not necessarily require the introduction of exogenous genes; rather, it can also be achieved by manipulating the expression of existing genes. To this end, recently, three types of nuclease-mediated gene editing tools have been developed to achieve efficient and precise genome targeting, with CRISPR/CRISPR-associated nuclease 9 (CRISPR/Cas9) being particularly notable. These tools enable techniques such as knock outs and site-specific insertion, which can be used to give pigs desired phenotypes, including optimal meat yield or quality and improved disease resistance. As a further improvement, base editors (BEs) and prime editors (PEs) derived from CRISPR/Cas9 technology allow for precise nucleotide substitutions in a programmable way without requiring a donor template. This capability may hold significant potential for improving genetics in pigs [5].

In 2022, Whitworth et al. [6] summarised advancements in germline editing efforts in swine and highlighted the numerous advantages these innovations offer for enhancing animal agriculture. Given the progress in research and the future trends associated with emerging CRISPR screening technologies, particularly in discovering new targets for pig disease resistance and recent significant applications in pig molecular breeding, motivates another review that focusses on these developments. This is especially relevant in the context of today’s efforts to strengthen and revitalise agriculture.

**Fig. 1:**
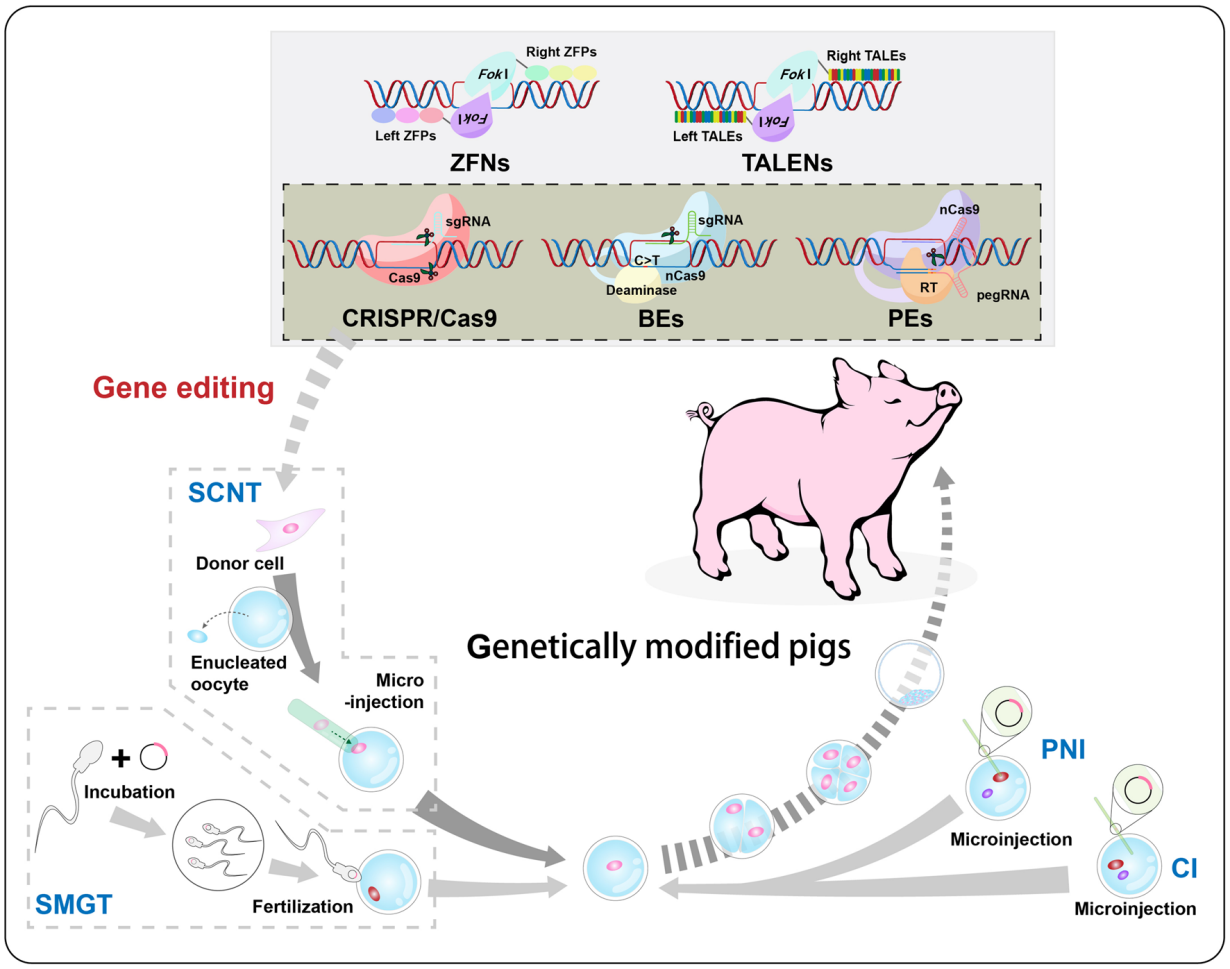
Advances in biotechnologies related to molecular breeding in swine. PNI, a transgenic DNA construct is directly microinjected into the pronucleus of a fertilised egg. CI, microinjecting the exogenous DNA into the cytoplasm of the fertilised egg. SMGT, involving co-incubation of sperm with exogenous DNA, followed by transferring the exogenous DNA into the oocyte at fertilization. SCNT, the nucleus of the donor somatic cell is transferred into the enucleated oocyte. The above methods are previous transgenic techniques to obtain genetically modified pigs. ZFNs (ZFPs are responsible for binding of specific DNA sequences and *Fok*I dimer performs cleavage), TALENs (the specific recognition ability of TALEs with the cleavage ability of *Fok*I dimer), and CRISPR/Cas9 (Guided by sgRNA, Cas9 binds to the target site and mediates cleavage) are gene editing techniques achieved by mediating DNA double-strand breaks. BEs, the sgRNA guides the complex of Cas9 variant and deaminase to the target site, where the deaminase deamidates the specified base to achieve the desired modification. PEs, fusing nCas9 with an engineered RT to achieve targeted substitutions, insertions and deletions guided by pegRNA. PNI, pronuclear microinjection; CI, cytoplasmic microinjection; SMGT, sperm-mediated gene transfer; SCNT, somatic cell nuclear transplantation; ZFNs, zinc finger nucleases; ZFPs, zinc finger proteins; *Fok*I, a type IIS restriction enzyme; TALENs, transcription activator-like effector nucleases; TALEs, transcription activator-like effectors; CRISPR/Cas9, clustered regularly interspaced short palindromic repeats/ CRISPR-associated nuclease 9; sgRNA, single guide RNA; BEs, base editors; nCas9, Cas9 nickase; PEs, prime editors; RT, reverse transcriptase; pegRNA, prime editing guide RNA

**Fig. 2:**
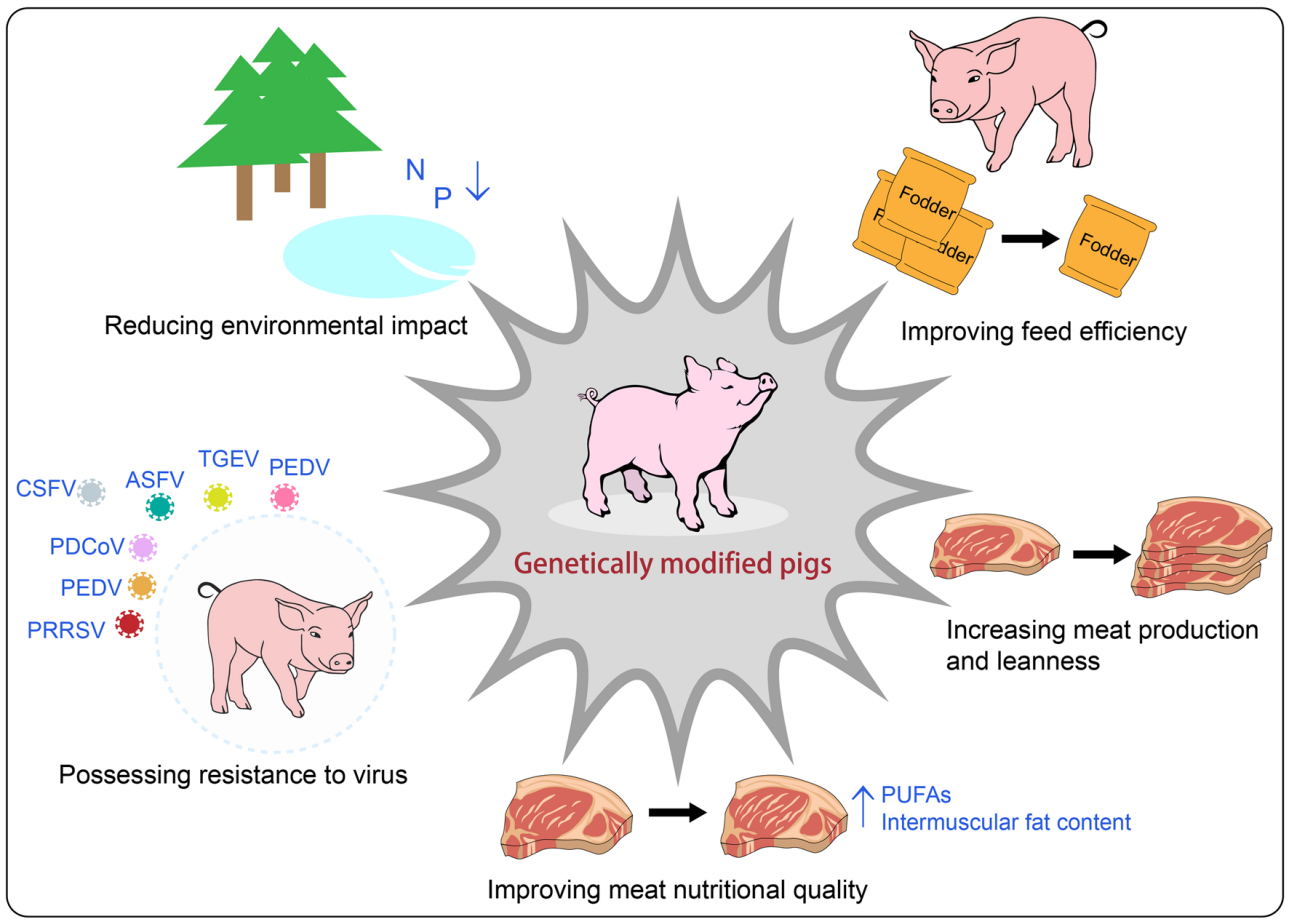
Applied aspects of molecular breeding in swine. PUFAs, polyunsaturated fatty acids; PRRSV, porcine reproductive and respiratory syndrome virus; PEDV, porcine epidemic diarrhea virus; PDCoV, porcine delta coronavirus; CSFV, classical swine fever virus; ASFV, African swine fever virus; TGEV, transmissible gastroenteritis virus

In this review, we systematically summarise and discuss the development trajectory (Fig. 1) and applications (Fig. 2) of relevant biotechnologies for gene editing in pigs. We focus on recent progress in the CRISPR high-throughput screening platform, particularly in identifying host factors associated with porcine viral replication. Additionally, we address the challenges and concerns related to advancing genetic improvement in swine. To help readers quickly grasp essential information about various genetically modified pigs and CRISPR screening applications, our article includes a tabular presentation that summarises each section.

## Advances in biotechnologies for the molecular breeding of pigs

Forty years ago, it was challenging to obtain new pig breeds swiftly and in a targeted manner to meet specific demands. However, with ongoing advancements in biotechnology, transgenic techniques, and gene editing technologies have become increasingly sophisticated. These innovations not only offer an ideal experimental framework for gene expression, regulation, and genetic research, but also provide a fast and effective way for targeted improvement of organisms and molecular breeding.

### Transgenic technology and pig breeding

Transgenic pigs are genetically modified lines created by transferring an exogenous gene into either germ cells or somatic cells. This method allows the exogenous gene to integrate into the porcine genome, enabling its expression. Since the first transgenic pig that was produced in 1985 [7], scientists have developed and applied various techniques to create transgenic pigs. The methods can be categorised into two main types: transferring exogenous genes into germ cells, which can be achieved through pronuclear microinjection, cytoplasmic microinjection, and sperm-mediated gene transfer, or transferring genes into somatic cells. These methods will be described in the following.

1. Pronuclear microinjection (PNI)

Pronuclear microinjection was once regarded as the traditional method for producing transgenic pigs. In this technique, a transgenic DNA construct is directly microinjected into the pronucleus of a fertilised egg, allowing it to be randomly integrated into the porcine genome. The first genetically modified pig was created using this method [7]. Over the next three decades, scientists successfully generated a variety of transgenic pigs by this approach [8]. However, the efficiency of producing transgenic offspring by PNI is only about 1% (number of transgenic pigs/number of injected embryos transferred), which necessitates the use of a large number of pronuclear stage embryos to be successful [9]. The number of embryos needed to produce a positive transgenic pig cannot be generalised, as it varies based on the type and size of the gene injected and the purity of the DNA [9]. Moreover, the application of PNI is limited due to the high operational demands and the mechanical damage caused by injecting into the pronucleus, which can negatively impact the normal growth of cells.

2. Cytoplasmic microinjection (CI)

Porcine-fertilised eggs have lower clarity, which often necessitates centrifugation before performing PNI. To simplify the procedure and minimise mechanical damage, Garrels et al. [10] injected the *Sleeping Beauty* (*SB*) transposon carrying *Venus fluorophore* into the cytoplasm of porcine fertilised eggs, successfully producing transgenic pigs. In another study, Li et al. [11] demonstrated that over 8% of the injected embryos developed into transgenic pigs using CI. The timing of exogenous gene injection is crucial, as the gene that enters the cytoplasm is translocated into the nucleus during prokaryotic membrane fusion [12].

3. Sperm-mediated gene transfer (SMGT)

The ability of spermatozoa to capture exogenous DNA was reported many years ago [13] but it was not until 1989 that this discovery was applied to the production of transgenic animals [14]. SMGT involves co-incubating sperm with exogenous DNA and then transferring the exogenous DNA into the oocyte during fertilization. For instance, Lavitrano et al. [15] modified the technique to create transgenic pig lines that express the human decay accelerating factor. In another study, researchers successfully produced multi-gene transgenic pigs by introducing three fluorescent reporter genes simultaneously using SMGT [16]. The efficiency of this method ranges from 5 to 60% [17]. Unlike microinjection, it does not require expensive equipment or tedious steps; it only requires artificial fertilisation of sperm cells that have interacted with exogenous DNA.

4. Somatic cell nuclear transplantation (SCNT)

The birth of Dolly, the cloned sheep, marked the beginning of mammalian SCNT technology [18]. In this method, the exogenous gene is transfected into somatic cells rather than into germ cells. Although the efficiency of genetically modifying somatic cells in livestock is only between 0.5 and 1% [19], positive cell screening can be performed in vitro. After selection and identification, the nuclei of these genetically modified somatic cells are transferred into mature denucleated oocytes, leading to the production of transgenic pigs through embryo transfer. The transgene positivity rate in pigs has significantly improved due to pre-screening. The combination of SCNT technology with subsequent development of gene editing tools provides a powerful platform for rapid and efficient molecular breeding of pigs.

### Gene editing technology and pig breeding

During the production of genetically modified pigs using the methods mentioned, integration of exogenous genes is primarily facilitated by viral vectors or DNA transposons, such as *SB* and *piggyBac* (*pB*). However, the randomness and instability of gene integration remain significant concerns. The recent emergence of gene editing technology, which enables precise modifications of genes, has demonstrated great potential for improving the genetics of pigs, elevating transgenic technology to a new level. The different tools available for gene editing are described in the following.

1. Zinc finger nucleases (ZFNs)

ZFNs were the first generation of gene editing tools. They consist of a specific DNA binding domain and a DNA cleavage domain. The recognition of the target site is carried out with 3–4 tandem Cys_2_-His_2_ zinc finger proteins (ZFPs) located at the N-terminal region, with each zinc finger unit recognising three bases. The DNA cleavage domain, found at the C-terminus, is derived from *Fok*I (a type IIS restriction enzyme) [20].

Upon design, the two ZFNs bind to their respective target DNA strands in, respectively, the 5′-3′ and 3′-5′ directions. When the two ZFNs bind to the target sequences that are located 5–7 bases apart, *Fok*I monomers can form a dimer and introduce DNA double-stranded breaks (DSBs) [21]. However, the vectors of ZFNs are complicated to construct and ZFPs have a weak affinity for the target site. In addition, the ZFNs-mediated shearing process is susceptible to forming heterodimers, which can lead to off-target effects and not all combinations of DNA triplet bases have corresponding zinc finger structures available [22].

Recently, Paschon et al. [23] developed an expanded set of ZFN architectures, allowing for the possibility of skipping bases between target triplets in otherwise adjacent fingers. These new architectures significantly enhanced the options for matching ZFPs to the target site and improved the accuracy of ZFNs, allowing for highly precise editing of specific genomic loci.

2. Transcription activator-like effector nucleases (TALENs)

The core of TALENs technology is the transcription activator-like effector (TALE), a natural protein secreted by *Xanthomonas* [24]. TALE regulates transcription by binding to the promoter of specific genes that contributes to colony formation in bacteria. Researchers have combined the specific recognition capability of TALE with the cleavage ability of *Fok*I to develop the second generation of gene editing tools, known as TALENs [25].

The tandem repeats in the central domain of TALE are responsible for its specific binding to the target DNA and these repeats are highly conserved, with each repeat unit typically consisting of 33 to 35 amino acids. Amino acids 12 and 13 in each repeat of the protein are highly variable and are known as repeat variable di-residues (RVDs) [26]. Different RVDs can recognise different bases, with NN, NI, HD, and NG RVDs showing strong preferences for G, A, C, and T, respectively [27].

When a TALE protein binds to its target site, *Fok*I cleaves DNA similarly to ZFNs. Compared to ZFNs, TALENs offer broader and more flexible targeting abilities and exhibit a lower off-target rate due to their highly specific binding to DNA target sites and recognition of longer DNA sequences. Despite their advantages, TALENs still have significant drawbacks, including high cost, time consumption, partially off-target effects, and cytotoxicity. Therefore, further improvements in convenience, accuracy, and safety are necessary.

3. CRISPR/Cas9

CRISPR/Cas is an adaptive immune system found in bacteria and archaea [28]. It consists of three major types: Type I, Type II, and Type III [29]. Each type features its own characteristic protein(s). The Type I and Type III systems require multiple Cas proteins to function together, whereas the Type II system relies on a single, large Cas9 protein. This has become the most widely used gene-editing tool, known as CRISPR/Cas9.

The Cas9 protein is a nuclease that contains two DNA-cleaving domains. The HNH domain cleaves the DNA strand complementary to CRISPR RNA (crRNA), while the RuvC-like domain cleaves the other strand. Guided by the crRNA and transactivating CRISPR RNA (tracrRNA) complexes, Cas9 binds to the target site and mediates DSBs [30].

To simplify this system, the crRNA and tracrRNA were merged to form a single guide RNA (sgRNA), which enables the Cas9 protein to target different locations by simply changing the sgRNA sequence [30]. The CRISPR/Cas9 system can simultaneously edit multiple targets, thereby facilitating flexible and convenient gene modification. This allows for the KO of multiple genes that influence a specific trait or for the deletion of long segments on a chromosome.

Since the first genome-cutting using CRISPR/Cas9 in mammalian cells in 2013 [31], this technology has shown great promise in various fields, including gene expression and regulation, agricultural breeding, the creation of animal disease models, gene therapy, and xenotransplantation [32–35]. Its simplicity and high efficiency contribute to its widespread application.

4. BEs and PEs

Recently, BEs based on CRISPR/Cas9 technology have been developed. These editors allow for single-base conversions without introducing DNA DSBs or requiring exogenous DNA donors. The two main types of BEs are the cytidine base editor (CBE) (C-to-T) and the adenine base editor (ABE) (A-to-G) [36, 37]. These BEs consist of a sgRNA, Cas9 variants, and specific deaminase proteins. The Cas9 variants are engineered to have partial or complete loss of cleavage activity through mutations in specific regions of the Cas9 nuclease domains. Examples of these variants include deactivated/dead Cas9 (dCas9) and Cas9 nickase (nCas9) [38]. The sgRNA directs the complex of the Cas9 variant and deaminase to the target site, where the deaminase deamidates the specified base to achieve the desired modification.

Compared to the first three generations of gene editing tools, BEs do not introduce DSBs. This is important because DSBs have been linked to potential risks such as chromosomal instability or the activation of oncogenes [39]. It is important to note that the use of deaminase in base editing still poses some safety risks [40].

Development PEs is based upon the BEs and are generated by fusing nCas9 with an engineered reverse transcriptase (RT). This technique allows for precise substitutions, insertions and deletions in the DNA [41]. During the editing process, a prime editing guide RNA (pegRNA, which is similar to sgRNA, but also contains a primer binding site and an RT template) guides the procedure [41]. nCas9 creates a nick in one strand of the DNA and then RT performs reverse transcription. During the DNA repair process, the unique properties of preferential 5’ flap excision and 3’ flap ligation facilitate the incorporation of the desired DNA sequence. The safety of using PEs, however, still lacks sufficient and comprehensive evidence and needs to be validated in future studies [42].

## Application of new biotechnologies in breeding for production traits in swine

### Increasing meat production and leanness

Pork is a significant source of meat for people in many countries and improving meat production and lean percentage has been a key goal in pig breeding efforts. As illustrated in Table 1, myostatin (*MSTN*) is one of the most researched genes aimed at promoting lean meat yield. Natural mutations in this gene have been extensively utilised in cattle and sheep breeding for over 50 years, resulting in a significant increase in lean meat production [43–45].

**Table 1:** Genetically modified pigs for increasing meat production and leanness

Target gene	Pig breeds	Modification type	Strategy	Method	Year	References
MSTN	Landrace	Exon 3 edit (homozygous, indels)	Gene editing (CRISPR/Cas9)	SCNT	2015	[48]
	Hubei White	Exon 3 edit (heterozygous KO)	Gene editing (CRISPR/Cas9)	SCNT	2016	[50]
	Erhualian	Exon 3 edit (homozygous KO)	Gene editing (CRISPR/Cas9)	SCNT	2017	[49]
	Duroc	Exon 3 edit (heterozygous, harboring the Belgian Blue mutation)	Gene editing (CRISPR/Cpf1-mediated HR)	SCNT	2019	[70]
	Chinese Bama	Exon 1 edit (Biallelic single-base insertion, frameshift mutations, and premature termination)	Gene editing (CRISPR/Cas9)	SCNT	2020	[51]
	Liang Guang Small Spotted	Monoallelic mutation in the signal peptide region (+ /PVD20H)	Gene editing (CRISPR/Cas9)	SCNT	2020	[52]
	Chinese Meishan	Exon 3 edit (homozygous, frameshift mutations, and premature termination)	Gene editing (TALENs)	SCNT	2022	[55]
	Large White	Exon 3 edit (homozygous, frameshift mutations, and premature termination)	Gene editing (TALENs)			
	Large White	Exon 1 edit (homozygous, frameshift mutations, and premature termination)	Gene editing (CRISPR/Cas9)			
IGF2	Chinese Bama	Indels (intron 3–3072 site)	Gene editing (CRISPR/Cas9)	CI	2018	[60]
	Liang Guang Small Spotted	Indels (intron 3–3072 site)	Gene editing (CRISPR/Cas9)	SCNT	2019	[61]
	Chinese Bama	*IGF2* intron 3 G3072A; CD163 R312Stop; MSTN Q218Stop	Gene editing (CBE)	CI	2022	[63]
	Liang Guang Small Spotted	Intron 3 C3071T	Gene editing (CBE)	SCNT	2023	[62]
	Chinese Bama	*IGF2* intron 3–3072 site indels; *ANPEP* KO; *CD163* KO; *MSTN* KO	Gene editing (Cas12iMax)	SCNT	2023	[64]
FBXO40	Chinese experimental mini	Exon 4 edit (homozygous, indels)	Gene editing (CRISPR/Cas9)	SCNT	2018	[66]
UCP1	Chinese Bama	Insert mouse *Ucp1* controlled by adipose tissue-specific adiponectin promoter into the porcine endogenous *UCP1* locus	Gene editing (CRISPR/Cas9-mediated knock-in)	SCNT	2017	[69]

The MSTN protein negatively regulates the proliferation and differentiation of skeletal muscle cells during both pre- and postpartum stages [46, 47] and, thus, various research teams have employed different editing strategies to disrupt *MSTN* in several pig breeds. Pigs with a successful MSTN disruption all displayed distinct phenotypic traits of increased muscle mass, including clearly visible intermuscular boundaries [48], broader backs and hips [49], and a higher number of muscle fibres [50–52].

Some studies have shown that a mutation in porcine *MSTN* exon 3 (which replicates a naturally occurring mutation in Belgian blue cattle) has led to a congenital hindlimb weakness syndrome in Western commercial breeds, such as the high-lean Large White and Landrace pigs [53, 54]. In contrast, targeting *MSTN* exon 3 in Chinese Indigenous high-fat Meishan pigs did not result in this pathologic phenotype [55]. Additionally, a recent study found that this issue could be resolved by editing *MSTN* exon 1 in the Western Large White and Landrace breeds, which may be related to the dimerization of MSTN [55].

Fan et al. [49] also provided extensive long-term and multidomain datasets for various *MSTN*-edited breeds. In addition to the increased lean meat production, there made several noteworthy observations: (i) *MSTN*-edited pigs had a comparable feed conversion ratio (FCR) to wild type (WT) controls due to reduced energy expenditure from exercise, although their maintenance energy consumption was greater; (ii) *MSTN*-edited sows did not experience dystocia or significant changes in litter size; (iii) *MSTN*-edited pork exhibited improved tenderness, higher protein, lower fat content, and an higher level of polyunsaturated fatty acids (PUFAs). These nutritional improvements are likely beneficial to human health, particularly for patients with cardiovascular diseases [55].

Insulin-like growth factor 2 (IGF2) is a crucial growth factor that supports growth and development in mammals during both fetal and postnatal stages. In mice, either a deficiency or overexpression of *Igf2* in mice has been linked to growth abnormalities [56, 57]. In pigs, quantitative trait locus (QTL) analysis identified a natural quantitative trait nucleotide (QTN) variant in intron 3 of the *IGF2* gene, specifically c.3072G>A [58]. In Western pig breeds, this substitution of the G at position 3072 in *IGF2* intron 3 to A disrupts the binding of the transcriptional repressor ZBED6. As a result there is an increase in *IGF2* expression in skeletal muscle, leading to improved lean meat yield [58, 59].

Certain indigenous pigs in China are known for their exceptional meat quality, particularly in intermuscular fat content, but tend to have slower rates of lean muscle growth. As a result, some researchers have explored ways to accelerate the rate of lean meat production and increase muscle mass by targeting the site in *IGF2* that binds with ZBED6 in Chinese native pigs. Some researchers have utilised CRISPR/Cas9-mediated non-homologous end joining to make indels at the *IGF2* 3072 site [60, 61], while others have employed BEs to create precise point mutations, such as C3071T [62] and G3072A [63]. These cloned pigs demonstrate enhanced growth performance and better muscle quality, which resulted from hypertrophy of the muscle fibres rather than hyperplasia [62]. Additionally, two research teams have edited multiple genes in a single step to simultaneously improve growth performance and resistance to infectious disease in Bama pigs [63, 64].

In 2018, Zou et al. [65] focussed on a gene restricted to muscle expression, *Fbxo40*, whose null mutation in mice results in a muscle hypertrophy phenotype. Using the CRISPR/Cas9 system, they targeted exon 4 of the gene to produce *FBXO40* KO pigs [66]. They found no differences in the relative number of myofibers between the *FBXO40* KO pigs and WT controls. However, the percentage of myofibers with a larger cross-sectional area was significantly greater in *FBXO40* KO pigs. Specifically, the average cross-sectional area of myofibers increased by 32.7% and carcass leanness improved by 4%. They also observed that the deficiency of FBXO40 led to a decrease in meat redness and an increase in shear force, which is consistent with the muscle hypertrophy phenotype. Notably, they found no significant effects on other meat quality traits such as pH, drip loss, fat content, moisture, and protein content [66].

Uncoupling protein 1 (UCP1) is a critical component involved in thermogenesis and plays a vital role in regulating energy homeostasis [67]. However, pigs lack functional UCP1 protein, which makes them more prone to fat deposition [68]. Zheng et al. [69] established adipose tissue-specific expression of mouse *Ucp1* using CRISPR/Cas9-mediated homologous recombination (HR). Ectopic expression of *Ucp1* significantly reduced fat accumulation and increased the percentage of lean meat in carcasses. Furthermore, these *Ucp1* knock-in pigs demonstrated an improved ability to maintain body temperature in cold conditions, although their physical activity and daily energy expenditure remained unchanged. These cloned pigs represent a promising breeding resource for enhancing lean meat production, improving pig welfare, and minimising economic losses caused by cold exposure [69].

### Improving nutritional quality of pork

As living standards have improved, people's demand for pork has evolved from simply needing to eat to eating well and seeking healthier options. As shown in Table 2, PUFAs are essential nutrients for human development and health. However, since mammals generally lack sufficient fatty acid desaturase, these PUFAs must be obtained through diet [71].

**Table 2:** Genetically modified pigs for improving nutritional quality of pork

Target gene	Pig breeds	Modification type	Strategy	Method	Year	References
FAD2	NA	Expression of *FAD2* gene from Spinach	Transgene	PNI	2004	[8]
fat-1	NA	Expression of humanised *fat-1* gene	Transgene	SCNT	2006	[76]
	Large White	Expression of *fat-*1 gene	Transgene	SCNT	2014	[77]
	Songliao Black	The *fat-1* gene was inserted into porcine Rosa 26 locus	Gene editing (CRISPR/Cas9-mediated knock-in)	SCNT	2018	[78]
	Chinese Laiwu	Co-expression of *fat-1* and *fat-2*	Transgene	SCNT	2019	[79]
PGC1α	Large White	Overexpression of *PGC1α* gene in muscle	Transgene	SCNT	2016	[83]
	Large White	Skeletal muscle-specific overexpression of *PGC1α*	Transgene	SCNT	2017	[82]
PPARγ	Large White	Muscle-specific overexpression of *PPARγ*	(1) Transgene (2) Gene editing (CRISPR/Cas9-mediated knock-in)	(1) SCNT (2) SCNT	2021	[84]

*NA* Not available

In 2004, Saeki et al. [8] created transgenic pigs that carry the *FAD2* gene (encoding fatty acid desaturase 2) from spinach. As a result, the linoleic acid content in the white adipose tissue of transgenic pigs was 20% higher than that in WT pigs. PUFAs are categorised into two categories: n-3 and n-6 PUFAs. n-3 PUFAs are primarily found in marine products and are primarily associated with various health benefits related to diseases such as neurodegenerative disorders, cardiovascular issues, inflammatory conditions, and cancer [72–74]. In contrast, n-6 PUFAs can promote inflammation [75]. Pigs lack the enzyme necessary to convert n-6 PUFAs into n-3 PUFAs.

As the most widely consumed meat in the world, pork can serve as an excellent source of n-3 PUFAs, particularly for individuals who consume few marine products. In 2006, Lai et al. [76] created transgenic pigs that express the humanised *Caenorhabditis elegans* gene, *fat-1*. This gene encodes a delta-15 desaturase, which facilitates the conversion of n-6 PUFAs into n-3 PUFAs. As a result, the *fat-1* transgenic pigs produced high levels of n-3 PUFAs and exhibited a significant decrease in the ratio of n-6 to n-3 fatty acids in tail samples.

Subsequently, Zhou et al. [77] confirmed the gene’s effectiveness in enhancing n-3 PUFAs in F1 transgenics offspring that carried the *Caenorhabditis briggsae fat-1* gene. With the development of gene editing tools and the challenges associated with random integration, Li et al. [78] utilised CRISPR/Cas9-mediated HR to insert the *fat-1* gene into the porcine Rosa26 locus. This modification led to a significant increase in the levels of n-3 PUFAs and a notable decrease in the n-6 PUFAs to n-3 PUFAs ratio, which changed from 9.36 to 2.12. These transgenic pigs were found to synthesise n-3 PUFAs from dietary sources rather than from endogenously produced n-6 PUFAs [78]. This was primarily due to the absence of the *fat-2* gene (encoding delta-12 desaturase), the enzyme responsible for converting internal oleic acid to linoleic acid.

Tang et al. [79] successfully created transgenic pigs that co-express *fat-1* and *fat-2* genes using SCNT technology. This advancement addresses the gap in the de novo n-3 PUFAs biosynthesis pathway.

Meat colour, muscle fibre types, and intermuscular fat content are key areas in pork quality research. Peroxisome proliferator-activated receptor gamma coactivator 1α (PGC1α) is a transcriptional co-activator that regulates mitochondrial biosynthesis and respiration [80]. This protein has been shown to play a role in determining muscle fibre types [81]. In two studies, specific overexpression of *PGC1α* in skeletal muscle led to increased mitochondrial respiration and fatty acid oxidation [82, 83]. This change resulted in the conversion of fast glycolytic fibres into slow and oxidative fibres, ultimately contributing to greater red meat production.

In addition, Gu et al. [84] created transgenic pigs that overexpress *PPARγ* (encoding peroxisome proliferator-activated receptor gamma), specifically in skeletal muscle. This genetic modification increased the intermuscular fat content by promoting adipocyte differentiation, while the lean meat ratio was unchanged in *PPARγ* compared to WT pigs. Additionally, the targeted overexpression of *PPARγ* in skeletal muscle enhanced the formation of oxidative fibres by activating the oxidative metabolism of fatty acids and the respiratory chain [84].

### Improving feed efficiency and reducing environmental impacts of pork production

Feed costs represent the most significant expense in pig production, typically accounting for two-thirds of total production costs. To reduce these costs, researchers have implemented various strategies to increase the uptake and absorption of nutrients from diets, which can lead to better efficiency and help mitigate environmental emissions. A common approach is to add engineered digestive enzymes to the pigs’diets to improve nutrient availability [85]. However, the effectiveness of these enzyme preparations can be influenced by multiple factors during production [86]. Additionally, these engineered enzymes must be resilient enough to withstand the different pH conditions encountered in vivo and resist degradation by intestinal proteases [6]. Another promising strategy is the natural expression of digestive enzymes in pigs through transgenic technology (Table 3).

**Table 3:** Genetically modified pigs for improving feed efficiency and reducing environmental impact

Target gene	Pig breeds	Modification type	Strategy	Method	Year	References
Phytase	Yorkshire	Expression of the *E. coli* appA phytase gene, driven by the parotid secretory protein promoter	Transgene	PNI	2001; 2013	[87, 88]
Cellulase	Duroc × (Yorkshire × Landrace)	Expression of the fungal cellulase gene, driven by the porcine pancreatic amylase promoter	Transgene	PNI	2015	[89]
β-glucanase, xylanase and phytase	Duroc	Simultaneously expressing three microbial enzymes, β-glucanase, xylanase, and phytase in the salivary glands	Transgene	SCNT	2018	[92]
β-xylanase	Duroc	Expressing the *xynB* from *Aspergillus Niger* CGMCC1067 in the parotid gland	Transgene	SCNT	2019	[91]
β-glucanase	Landrace	Specifically expressing β-glucanase gene in the intestine	Transgene	SCNT	2019	[90]

Phytic acid serves as a phosphorus storage molecule in plants but pigs cannot digest it because they lack the enzyme phytase. As a result, most of the organic phosphorus is excreted in faeces, contributing to environmental pollution. To meet the nutritional needs of pigs, inorganic phosphates must be added to their diets. In an initial study to address this shortcoming in pigs, Golovan et al. [87] obtained transgenic pigs that express the bacterial phytase in their salivary glands using PNI. This salivary phytase was shown to function effectively under the low pH conditions of the pig’s digestive tract, allowing for the nearly complete digestion of dietary phytate phosphorus, which reduces faecal phosphorus output by up to 75% [87]. Follow-up monitoring over nine generations confirmed that the integration site of this transgene remained stable [88].

Non-starch polysaccharides (NSP) are a type of molecule found in feed ingredients that cannot be digested because the body lacks endogenous NSP-degrading enzymes. For instance, arabinoxylans and β-glucans, which are present in the cell walls, create a barrier that hinders hydrolysis and absorption of nutrients. Additionally, NSP can slow down the diffusion of nutrients into the intestines by reducing the contact between digestive enzymes and the chyme. Several teams have developed transgenic pigs that express a single gene for digesting NSP, including fungal cellulase [89], β-glucanase [90], and xylanase [91]. While these modifications have resulted in some partial improvements, a single digestive enzyme gene alone is inadequate for effectively breaking down various NSPs and phytate present in feedstuff.

Zhang et al. [92] produced genetically modified pigs that simultaneously express β-glucanase, xylanase, and phytase in their salivary glands. These transgenic pigs demonstrated an 11.5 to 14.5% increase in FCR and a 23.2 to 45.8% reduction in faecal nitrogen and phosphorus outputs. In their review, Whitworth et al. [6] provided a more detailed summary of various studies focused on creating genetically engineered pigs with enhanced digestion.

In addition to the points mentioned above, it is important to highlight the galactose-α-1,3-galactose (α-Gal) deficient pig, which is the first genetically modified pig approved by the U.S. Food and Drug Administration for both human consumption and medical applications [93]. In humans, the gene (*GGTA1*) responsible for producing alpha 1,3 galactosyltransferase is a non-functional pseudogene. As a result, consuming meat that contains α-Gal can trigger an allergic reaction in some individuals [94]. Several research teams have disrupted the porcine *GGTA1* gene using either HR or gene editing technologies, successfully creating pigs that lack the α-Gal epitope [95–99]. This development provides safe meat for people with α-Gal syndrome. Additionally, recognition of the α-Gal epitope by natural antibodies is a significant factor in immune rejection [100]. Therefore, blocking the formation of the α-Gal epitope can effectively prevent hyperacute rejection in xenotransplantation. Thus, the α-Gal deficient pig holds significant promise for medical applications, particularly in drug production and organ provision for xenotransplantation. Using specially genetically modified pigs as donors for xenografts could help address the ongoing shortage of human organs.

## Application of new biotechnologies in breeding for disease resistance in swine

Pig farming plays a crucial role in the global livestock industry, but it is often seriously threatened by viral diseases. Notable among these are porcine reproductive and respiratory syndrome (PRRS), African swine fever (ASF), classical swine fever (CSF), porcine epidemic diarrhoea (PED), and transmissible gastroenteritis (TGE). The rapid spread of these viruses has been exacerbated by high-density industrialised farming practices. Additionally, the high rates of recombination and variation among viral strains make the prevention and control of these epidemics difficult.

In addition to using vaccinations to combat infectious swine diseases, breeding pigs that are resistant to these diseases through genetic modification has emerged as a key area of research. Gao et al. [101] recently provided a summary of molecular breeding techniques aimed at enhancing disease resistance in various livestock species, including pigs, cattle, cows, sheep, goats, and chickens. They emphasised the crucial role of breeding disease-resistant animals in promoting the sustainability and productivity of global food systems. Our summary of the use of genetic modification to breed resistant pigs is in Table 4 and will be described in detail in the following. Since various strains of the same virus may represent different disease resistance traits, we have included the strains used for the in vivo virus challenges used in the referenced studies in the table to assist in further analysis and exploration.

**Table 4:** Genetically modified pigs for disease resistance

Virus	Target	Pig breeds	Modification type	Strategy	Method	Strain used for in vivo challenge	Year	References
PRRSV	*CD169*	Landrace	KO	Transgene (HR-mediated KO)	SCNT	PRRSV-2 KS-06 (No effect)	2013	[120]
	*CD163*	Large White	KO (exon 7 edit, indels)	Gene editing (CRISPR/Cas9)	SCNT	PRRSV-2 NVSL 97–7895 (Completely resistant)	2014; 2016	[122, 123]
		Duroc	KO (exon 7 edit, indels)	Gene editing (CRISPR/Cas9)	SCNT	HP-PRRSV TP (Completely resistant)	2018	[125]
		Large White	*CD163* KO (exon 7 edit, indels); *APN* KO (exon 2 edit, indels))	Gene editing (CRISPR/Cas9)	SCNT	PRRSV-2 WUH3 (Completely resistant) TGEV WH-1 (Completely resistant); PDCoV CHN-HN-2014 (Decreased susceptibility)	2020	[126]
	CD163 SRCR5	Large White	CD163-SRCR5 KO	Gene editing (CRISPR/Cas9)	CI	PRRSV-1 BOR-57 (Completely resistant)	2017; 2018	[130, 131]
		Large White	CD163-SRCR5 KO	Gene editing (CRISPR/Cas9)	SCNT	HP-PRRSV JXA1 (Resistant but still infected)	2019	[132]
		NA	Substitution of CD163 SRCR5 with hCD163L1 SRCR8	HR	NA	PRRSV-1 SD13-15 (Completely resistant) PRRSV-2 NVSL 97–7895 (No effect) PRRSV-2 KS06-72109 (No effect)	2017	[133]
		Landrace	Substitution of CD163 SRCR5 with hCD163L1 SRCR8	Gene editing (CRISPR/Cas9)	SCNT	HP-PRRSV JXA1 (Resistant but still infected)	2019	[134]
	A portion of PSTII in CD163	NA	Deletion of exon 13 that encodes the first 12 amino acids of PSTII domain	Gene editing (CRISPR/Cas9-mediated HR)	SCNT	PRRSV-2 NVSL (Completely resistant)	2024	[136]
	Key amino acids of CD163 SRCR5	Large White	41 amino acids (481–521) KO	Gene editing (CRISPR/Cas9)	SCNT	HP-PRRSV JXA1 and MY (Completely resistant)	2019	[137]
		Large White	CD163 R561A	Gene editing (CRISPR/Cas9)	SCNT	PAMs isolated from the cloned pigs: PRRSV-2 WUH3 (Resistant but still infected)	2023	[138]
CSFV	CSFV shRNA	Large White	shRNA inserted at porcine Rosa26 locus	Gene editing (CRISPR/Cas9-mediated knock-in)	SCNT	CSFV Shimen (Reduced CSFV infection)	2018	[145]
	*RSAD2*	Large White	*RSAD2* inserted at porcine Rosa26 locus	Gene editing (CRISPR/Cas9-mediated knock-in)	SCNT	CSFV Shimen (Reduced CSFV infection)	2019	[148]
	*PCBP1*	Large white	KO	Gene editing (CRISPR/Cas9)	SCNT	CSFV Shimen (Reduced CSFV infection)	2022	[149]
ASFV	*CD163*	Large White	KO	Gene editing (CRISPR/Cas9)	SCNT	ASFV Georgia 2007/1 (No effect)	2017	[155]
	*RELA*	Large White	Substitution with 3 or 2 of the amino acid variations from the warthog RELA orthologue	Gene editing (ZFN-mediated HR)	CI	ASFV Ken05/Tk1 (No effect))	2016; 2020	[157, 158]
Porcine corona-viruses	*APN*	Large White	KO (exon 2 edit, indels)	Gene editing (CRISPR/Cas9)	CI	TGEV Purdue (Completely resistant) PEDV KS13-09 (Retained susceptibility to infection)	2019	[167]
		Large White	KO (exon 2 edit, indels)	Gene editing (CRISPR/Cas9)	SCNT	PEDV field isolate from South China (No effect)	2019	[166]
		Large White by Landrace cross	KO (exon 2 edit, indels)	Gene editing (CRISPR/Cas9)	SCNT	TGEV JS2012 (Completely resistant) PEDV AH2012/12 (No effect)	2019	[165]
		Large White	Residues 717 to 839 were replaced with the homologous murine APN	Gene editing (CRISPR/Cas9-mediated knock-in)	SCNT	Porcine kidney epithelial cells isolated from the gene-edited pigs: TGEV (Resistant but still infected)	2023	[169]

*NA* Not available

### PRRS

PRRS is characterised by reproductive failure in pregnant sows, respiratory diseases in young pigs, reduced growth performance, and increased mortality rates [102]. The causative agent of PRRS is the PRRS virus (PRRSV), which is a small enveloped RNA virus belonging to the mammalian arterivirus group. The genome of PRRSV consists of a single-stranded, positive-sense RNA that is approximately 15 kb in size [103]. PRRSV strains have been classified into two genotypes: European (type I) and North American (type II) [104]. The virus exhibits extensive genetic variation and recombination, which often leads to the emergence of various virulent and pathogenic strains [105–107]. Modified-live virus vaccines (MLVs) are the primary method used to prevent PRRS. However, these MLVs can regain virulence and often offer limited or no cross-protection against heterologous strains [108, 109]. As a result, researchers have extensively studied the development of genetically modified pigs that are resistant to various heterologous PRRSV infections.

The targeted cells for PRRSV are porcine alveolar macrophages (PAMs) in pigs [110]. Several cellular receptors that facilitate the entry of PRRSV into PAMs have been identified, including SIGLEC1 (CD169) [111, 112], CD163 [113], heparin sulfate (HS) [114], vimentin [115], myosin heavy chain 9 (MYH9) [116, 117], CD209 [118], and Siglec-10 [119]. Initially, Prather et al. [120] generated *CD169* KO pigs using HR and SCNT, which were then challenged with PRRSV. They, however, found no significant difference in virus replication between the *CD169* KO pigs and WT pigs.

Subsequently, researchers have focused on utilising CRISPR/Cas9 technology to disrupt CD163 or its functional domains to enhance resistance to PRRSV infection. CD163 is a macrophage-specific glycoprotein that belongs to the scavenger receptor cysteine-rich (SRCR) superfamily, which includes a diverse array of membrane proteins involved in recognising various ligands. Porcine *CD163* consists of 17 exons and includes a signal peptide and nine extracellular SRCR domains (each domain consists of 100–110 amino acid residues). Additionally, it contains two intra-domain segments known as proline serine threonines (PSTs), a transmembrane region, and one intracellular cytoplasmic tail [121]. Studies on targeting CD163, its functional domains, or its key amino acids are described in the following.

1. Targeting *CD163*

In 2014, Whitworth et al. [122] successfully created *CD163* KO pigs using CRISPR/Cas9 technology. These pigs were completely resistant to the type II PRRSV isolate NVSL 97–7895 [123]. The *CD163* gene-edited pigs did not exhibit any clinical signs, such as fever or respiratory issues, and showed no lung pathology, viremia, or antibody response after infection. This study first verified in vivo that CD163 is an essential receptor for PRRSV infection. Subsequent trials conducted by this research team further established that these maternal *CD163* gene-edited pigs can protect their fetuses from infection with PRRSV [124]. In 2018, Yang et al. [125] demonstrated that the KO of *CD163* in pigs also results in complete resistance to highly pathogenic PRRSV (HP-PRRSV TP) infections. In 2020, Xu et al. [126] successfully created cloned pigs with a double KO (DKO) of two known receptor proteins, CD163 and APN. The DKO pigs exhibited total resistant to both highly pathogenic PRRSV (HP-PRRSV WUH3) and the TGE virus (TGEV). Additionally, there were no significant differences in meat production or reproductive performance compared to the WT pigs but an increase in iron content in the muscle of DKO pigs was observed. This study demonstrated that multi-gene editing can be combined to achieve simultaneous resistance to various major porcine viruses. All of these studies established that targeting the *CD163* gene plays a key role in breeding PRRSV-resistant pigs.

2. Targeting the key domain of CD163

The transmembrane anchoring of the SRCR5 domain of CD163 and its interaction with PRRSV are essential for successful PRRSV infection [127, 128]. Considering that (i) CD163 plays significant biological roles, particularly in mediating systemic inflammation and regulating immune responses [129] and (ii) there are no specific functions associated with the SRCR5 domain aside from its involvement in PRRSV infection [128], researchers aimed to precisely edit this key domain of CD163. In 2017, Burkard et al. [130] successfully generated pigs with a deletion of exon 7 in the *CD163* gene (encoding the SRCR5 domain). The cloned pigs were healthy and the biological functions of CD163 were confirmed to be intact. Additionally, macrophages from the cloned pigs demonstrated full resistance to both type I and type II PRRSV in vitro. When challenged with type I PRRSV in vivo, these pigs exhibited no signs of infection, viremia, or antibody response [131]. However, research by Wang et al. [132] in 2019 indicated that, while the absence of the CD163 SRCR5 domain provides resistance to the HP-PRRSV JXA1 strain without compromising the biological functions of the CD163, some mild clinical symptoms were observed in the cloned pigs during the viral challenge.

Meanwhile, some researchers have expressed chimeric CD163 proteins by replacing the porcine CD163 SRCR5 domain with a homologous human CD163-like (hCD163L1) SRCR8 domain. The resulting cloned pigs demonstrated resistance to type I PRRSV, although they were still susceptible to type II PRRSV [133]. Additionally, these pigs were not entirely resistant to HP-PRRSV but they were able to significantly inhibit PRRSV replication by blocking virus uncoating and genome release [134]. In addition to the SRCR5 domain, Stoian et al. [135] identified several critical regions in CD163 that are essential for PRRSV infection in vitro, including the SRCR4/5 interdomain, exon 13 (which encodes a portion of PSTII), and a pentapeptide domain located in both SRCR5 and SRCR7. Salgado et al. [136] recently produced genetically modified pigs that lack the PSTII-domain-coding exon 13 of CD163, which allowed these pigs to completely resist type II PRRSV infection, without affecting the primary physiological functions associated with CD163 in vivo. Furthermore, the PAMs from the modified pigs demonstrated complete resistance to both type I and type II PRRSV in vitro.

3. Targeting key amino acids of CD163

To better target the portion of the CD163 SRCR5 domain associated with PRRSV infection, Guo et al. [137] deleted 41 amino acids (481–521) containing the ligand-binding pocket (LBP) in the CD163 SRCR5 domain. The gene-edited pigs exhibiting this modification were found to be completely resistant to two strains of type II PRRSV: the JXA1 and MY strains. This work provides valuable insight into identifying the crucial regions of the SRCR5 domain involved in viral infection. Recently, Xu et al. [138] discovered that a deletion of 40 amino acids (523–562) in the CD163 SRCR5 domain of immortalised PAMs also led to complete resistance against type II PRRSV infection. Moreover, macrophages isolated from cloned pigs carrying CD163 R561A demonstrated significantly lower susceptibility to PRRSV compared to WT pigs. However, CD163 R561 was not essential for the virus’s infection [138].

### CSF

CSF is a severe and highly contagious disease caused by the CSF virus (CSFV) in pigs. CSFV is an enveloped, single-stranded, positive-sense RNA virus that belongs to the *Pestivirus* genus within the *Flaviviridae* family. The clinical signs of CSFV infection include fever, haemorrhages, and convulsions, often resulting in high mortality rates [139]. The genome of CSFV is approximately 12.3 kb long and encodes a precursor polyprotein that consists of 3,898 amino acids. This polyprotein is further cleaved into four structural proteins (C, E^rns^, E1, and E2) and eight non-structural proteins (N^pro^, p^7^, NS2, NS3, NS4A, NS4B, NS5A, and NS5B) [140]. Several receptors have been identified to play key roles in the infection of CSFV. These receptors include HS, laminin receptor (LamR), and the complement regulatory protein (CD46), as well as attachment factors such as MER tyrosine kinase (MERTK), metalloproteinase domain-containing protein 17 (ADAM17), and Annexin 2 [141].

To control CSF, two main strategies are currently used: systematic prophylactic immunisation and the culling of infected animals [142]. Prophylactic immunisations consist of live attenuated vaccines, subunit vaccines, and chimeric pestivirus vaccines. These vaccines have significantly contributed to the prevention and control of CSF epidemics, as well as the eradication and purification of CSFV [143]. However, eliminating CSF in areas where the disease is endemic or reemerging remains a challenge [144]. Consequently, researchers have been exploring the modification of the porcine genome using gene editing tools to develop pigs that are resistant to CSFV.

In 2018, Xie et al. [145] combined RNA interference (RNAi) technology with CRISPR/Cas9-mediated HR to create transgenic pigs that produce short hairpin RNA (shRNA) targetting CSFV, which was inserted into the porcine Rosa26 safe harbour locus. In vivo viral challenge assays demonstrated that these cloned pigs could effectively limit CSFV replication and exhibited a reduced incidence of clinical signs and mortality associated with CSFV. Additionally, the resistance to CSFV was stably inherited in F1 pigs [145].

Following this, Lu et al. [146] discovered that anti-CSFV shRNA could integrate into the porcine miRNA-17-92 cluster using a CRISPR/Cas9-mediated knock-in strategy, demonstrating antiviral effects in vitro. Furthermore, RSAD2 (also known as viperin) has shown the capability to inhibit the proliferation of various DNA and RNA viruses [147]. In 2020, Xie et al. [148] inserted porcine *RSAD2* into the porcine Rosa26 locus, which allowed for stable and efficient expression of the RSAD2 protein. Viral challenge studies conducted in vitro revealed that overexpressing porcine *RSAD2* effectively reduced infections from CSFV and pseudorabies virus (PRV), but did not impact replication of PED virus (PEDV) or porcine circovirus 2 (PCV2).

Qi et al. [149] focused on a crucial protein, PCBP1, that mediates the interactions between host cells and CSFV. Their study found that gene-edited pigs lacking the *PCBP1* gene exhibited normal birth weight, development, and reproductive abilities. Primary cells isolated from both F0 and F1 pigs showed a significant reduction in CSFV infection. Additionally, the antiviral effect resulting from the deficiency of PCBP1 was closely associated with activation of type I interferon [149].

### ASF

ASF is a deadly hemorrhagic disease caused by the African swine fever virus (ASFV), which has a nearly 100% lethality rate [150]. Currently, there is no effective commercial vaccine or treatment for ASF, so its control primarily depends on early diagnosis and swift eradication efforts [151]. ASFV is a large, complex virus with double-stranded DNA, and it is the only member of the family *Asfarviridae* [151]. The genome of ASFV ranges from 170 to 190 kb and can encode more than 150 proteins within infected cells [152]. This virus specifically targets monocytes and macrophages in both domestic and wild swine [153].

A previous study indicated that ASFV-infected macrophages exhibited increased levels of CD163 protein. Furthermore, the use of anti-CD163 monoclonal antibodies was found to inhibit ASFV infection and reduce the binding of viral particles to PAMs [154]. To determine whether CD163 serves as a potential or essential receptor for ASFV infection, Popescu et al. [155] conducted an experiment in which they infected *CD163* KO pigs and WT controls with the Georgia 2007/1 ASFV isolate. The results showed no significant differences in clinical symptoms, mortality rates, pathology or levels of viremia between the two groups. This indicates that CD163 is not required for ASFV infection.

In contrast to the acute and highly virulent nature of ASFV in domestic pigs, warthogs display weaker pathogenicity or may show no symptoms of infection at all. Palgrave et al. [156] identified three amino acid differences in the *RELA* gene and proposed that these variations may explain the different responses of the two species to ASFV infection. They then employed ZFN-mediated HR to create domestic pigs with either two or all three amino acids substituted from the warthog RELA orthologue but they were not able to resist ASFV completely [157]. However, some animals did exhibit a delay in the onset of clinical signs and a reduction in viral DNA in blood samples and nasal secretions [158]. Further studies are needed to understand the interaction between ASFV and its host, clarify the key factors that facilitate ASFV infection, and identify effective genetic targets for breeding ASFV-resistant pigs.

### Porcine coronaviruses

Coronaviruses belong to the subfamily *Coronavirinae*, within the family *Coronaviridae* and the *Nidovirales* order. They are responsible for respiratory and intestinal infections in both humans and various animal species [159]. These viruses are large, enveloped, positive-stranded RNA viruses that encode four main structural proteins (S, M, N, and E). The S glycoproteins give the virus a crown-like appearance and facilitate its entry into host cells [160, 161].

In pigs, the primary enteric coronaviruses include TGEV, PEDV, porcine delta coronavirus (PDCoV), and swine acute diarrhoea syndrome coronavirus (SADS-CoV). These viruses can cause severe diarrhoea, vomiting, and dehydration, particularly in neonatal piglets [162]. Mortality of newborn piglets infected with TGEV or PEDV can reach up to 100%. In contrast, PDCoV infection presents milder clinical symptoms and has a lower lethality rate (30 to 40%) compared to TGEV or PEDV infections [163].

An earlier study found that Aminopeptidase N (APN), a transmembrane ectoenzyme that is abundantly expressed on intestinal epithelial cells, serves as a primary receptor for TGEV [164]. APN facilitates invasion of host cells by TGEV by binding to the C-terminal domain of the S1 subunit of the TGEV S protein [160].

In 2019, several teams utilised CRISPR/Cas9 technology to generate APN-null pigs, which indicated that APN deficiency conferred resistance to TGEV infection, but not to PEDV infection [165–167]. It has been reported that residues 717–813 of porcine APN are essential for TGEV infection [168]. Recently, to further optimise the gene-editing strategy for porcine *APN*, Liu et al. [169] produced APN-chimeric pigs (residues 719–839 were replaced with the homologous murine APN) using a CRISPR/Cas9-mediated knock-in strategy. Porcine kidney epithelial cells isolated from the resulting F1-generation APN-chimeric pigs demonstrated effective resistance to TGEV infection and normal pregnancy rates and viability. Exogenous expression of porcine *APN* in refractory cells, such as Vero and BHK-21 cells, conferred susceptibility to PDCoV [170]. Furthermore, PAMs from *APN* KO pigs showed resistance to PDCoV infection [171]. These findings suggest that APN serves as a receptor for PDCoV infection.

Xu et al. [126] generated *CD163* and *APN* DKO pigs using CRISPR/Cas9 and the resulting cloned pigs displayed complete resistance to PRRSV and TGEV, as well as reduced susceptibility to PDCoV. This indicates that while APN is one of the receptors for PDCoV, it is not the only receptor involved in the infection.

### Challenges and concerns in advancing genetic improvement in swine

Transgenic and gene editing technologies have shown great promise in pig breeding for improving production traits and disease resistance. Unlike previous transgenic methods, gene editing strategies minimise the safety risks associated with random gene integration and significantly enhance the efficiency of gene modification. However, even with the precise gene editing tools available today, there remains a risk of unintended edits occurring in other locations within the genome.

Currently, our understanding of the major genes related to production traits and mechanisms of viral infections is still limited. Identifying additional editable genes with defined functions and clear regulatory networks is essential for advancing molecular breeding, primarily relying on developments in functional genomics and molecular biology. In addition, identifying the structural domains or amino acid residues that are crucial in major genes and then editing them with precision can help prevent adverse effects, such as disrupting normal physiological functions.

Few studies have performed long-term, multidomain analyses of genetically modified pigs. However, the real application and economic value of any trait depend on a systematic assessment of resource populations, not just on proof-of-concept animals. For instance, in molecular breeding aimed at improving meat production in swine, other important commercial traits such as FCR, pork quality, and fertility should also be evaluated in a long-term and comprehensive manner.

Currently, only two genetically modified traits have received regulatory approval for entry into the commercial market. One of these involves pigs altered for *CD163* [172], while the other involves α-Gal deficient pigs (https://www.fda.gov/news-events/press-announcements/fda-approves-first-its-kind-intentional-genomic-alteration-line-domestic-pigs-both-human-food). Social acceptance and regulatory policies regarding genetically modified pigs pose significant challenges to advancing genetic improvements in swine [173, 174] and other species.

A common concern among the public is that genetically modified animals are unnatural. While science can define the characteristics of a genetically modified animal, it cannot determine whether these animals should be commercialised from a public policy perspective. Furthermore, the safety assessment process for genetically modified animals is complex and obtaining approval can be slow. Even after receiving a safety certificate, additional commercially relevant certifications must be secured before genetically modified animals can be marketed.

## CRISPR genetic screens to identify host factors in porcine virus replication

In recent years, CRISPR genetic screening approaches have rapidly gained prominence. These approaches utilise the CRISPR/Cas9 system to create a vast number of mutant cells, followed by applying specific screening pressures to identify the relationship between certain phenotypes and genotypes [175]. This screening strategy addresses the limitations of traditional techniques, which often suffer from incomplete protein depletion and high off-target effects associated with RNAi screening. As a result, it offers an efficient, versatile, and large-scale platform for functional genomic screen [176, 177].

Several studies have employed this approach to uncover host factors critical for infection and replication of porcine viruses (Table 5). This not only enhances our understanding of the regulatory network involved in virus-host interactions and deepens our knowledge of the viral replication cycle but also provides new targets and strategies for developing disease resistance in swine. The primary aim of using CRISPR screens to identify host factors is to target infectious cell-lethal viruses, such as TGEV [178] and PRRSV [179]. For viruses that do not induce cytopathic effects, recombinant viruses that express fluorescent proteins can be employed to sort mutant cell populations based on fluorescence intensity. An example of such as virus is the vesicular stomatitis virus (VSV) [180].

**Table 5:** CRISPR screens to identify host factors in porcine virus replication

Virus	Library	Coverage of sgRNAs	Cells for screening	Screening rounds	Main candidates	Year	References
JEV	A porcine genome-scale CRISPR KO library	85,674 sgRNAs (targeting 17,743 protein-coding genes, 11,053 lncRNAs and 551 miRNAs) and 1,000 negative control sgRNA	PK15-Cas9	Four rounds (MOI = 0.3)	*SLC35B2*, *HS6ST1*, *B3GAT3*, *GLCE*, *EMC3*, *CALR*	2020	[181]
TGEV	A porcine genome-scale CRISPR KO library	85,674 sgRNAs (targeting 17,743 protein-coding genes, 11,053 lncRNAs and 551 miRNAs) and 1,000 negative control sgRNA	PK15-Cas9	Three rounds (MOI = 0.001)	*TMEM41B*	2021	[178]
	A focused CRISPR library screen	Targeting 79 top-ranked candidate host factors, 6–7 sgRNAs per gene		Five rounds (MOI = 1)			
PRV	A porcine CRISPR/Cas9 KO library (SsCRISPRko.v1)	83,381 sgRNAs (targeting 20,598 genes) and 1,000 non-targeting control sgRNAs	PK15	Two rounds (MOI = 0.5)	*SMS1*	2021	[183]
PDCoV	Two genome-scale CRISPR KO library	A library: 59,088 sgRNAs; B library: 55,007 sgRNAs (targeting a total of 20,081 genes)	LLC-PK1-Cas9	Five rounds (MOI = 0.1)	*TMEM41B*, *SLC35A1*, *SNX10*, *VOPP1*, *PCSK6*	2022	[184]
PRRSV	A porcine genome-scale CRISPR/Cas9 KO library	65,525 sgRNAs (targeting 13,720 protein-coding genes)	PK15-Cas9-CD163	Four rounds (MOI = 1)	*KXD1*, *VTN*, *UBB*, *PPP2CA*, *NDUFB3*, *MAP4K3*, *LGALS2*	2022	[179]
SADS-CoV	A human genome-wide CRISPR/Cas9 KO library	77,441 sgRNAs and 1,000 non-coding control sgRNAs	Huh7.5	Four rounds	*PLAC8*, *SCAP*	2022	[185]
VSV	An ISG (IFN-stimulated genes)-targeting CRISPR/Cas9 KO library	1,908 sgRNAs (targeting 5’ constitutive exons of 359 ISGs)	IBRS-2	Three rounds (MOI = 0.1)	*IRF9*, *IFITM3*, LOC100519082, *REC8*	2022	[180]
PEDV	A pooled African green monkey genome-scale CRISPR/Cas9 KO library	75,608 sgRNAs (targeting 18,993 protein-coding genes) and 500 non-targeting control sgRNAs	Vero E6-Cas9	Two rounds (first round: MOI = 0.00001; second round: MOI = 0.0001)	*TRIM2*, *SLC35A1*	2023	[186]
JEV	A CRISPR-Cas9-mediated cytosine base editing library	457 sgRNAs (targeting all exons and introns of *CALR*) and 63 negative control sgRNAs	PK15-BE4	One round (MOI = 0.1)	GGGG → AAAA in intron 2 of *CALR*	2023	[182]
PRRSV	A porcine genome-scale CRISPR-Cas9 KO library	NA	3D4/21-CD163-Cas9	Three rounds (MOI = 0.01)	*SMPDL3B*	2023	[187]
ASFV	A porcine CRISPR/Cas9 KO library (SsCRISPRko.v1)	83,381 sgRNAs (targeting 20,598 genes) and 1,000 non-targeting control sgRNAs	WSL	Four rounds (MOI = 0.3 or 0.5)	SLA-DMA, SLA-DMB, CIITA, RFXAP	2023	[188]

*NA* Not available

As an example, Zhao et al. [181] identified the key host factors necessary for Japanese encephalitis virus (JEV) infection using a porcine genome-scale CRISPR/Cas9 KO library. In their study, they used the lethality of JEV infection in PK15 cells as the screening criterion. After conducting four consecutive rounds of viral challenge, they screened out *EMC3* and *CALR*, which are involved in calcium homeostasis, along with four genes associated with heparan sulfate proteoglycans metabolism. Subsequently, Xiong et al. [182] performed a CRISPR/Cas9-mediated cytosine base editing screen that targets all exons and introns of *CALR* to identify variants that could inhibit JEV replication. A *CALR* variant that involved changing four consecutive G bases in intron 2 to four A bases was found to significantly inhibit JEV replication [182]. This finding is important for accurately editing non-coding regions to enhance antiviral capabilities. By combining genome-wide CRISPR/Cas9 KO screens with point-mutation libraries that target candidate genes, novel targets can be identified and precise, beneficial variants can be pinpointed. CRISPR screens, including CRISPR KO, point mutations, activation, and interference, can also help uncover functional genes related to other important traits, such as muscle growth and development, as well as intramuscular fat formation.

## Conclusions

Rapid breeding of pigs using transgenic and genome editing technologies is vital for the future of pork production and has significant potential to enhance animal health and welfare, while also contributing to human health and food security. Overall, gene manipulation can be categorised into three main approaches: (i) inactivation of endogenous genes (such as an edit for inactivation of *MSTN* to increase skeletal muscle mass); (ii) insertion of exogenous genes, including genes from other species (such as *fat-1* knock-in to increase PUFAs content) and specific synthetic sequences (like CSFV shRNA knock-in to confer disease resistance); and (iii) introducing a beneficial genetic sequence that already exists in the same species, equivalent to natural variation, by gene editing. The latter approach is safest and most socially acceptable.

CRISPR/Cas9 technology is currently the most widely method for gene editing. However, further applications of BEs and PEs, as well as the optimisation of gene editing systems, are essential to advance molecular breeding. One of the key challenges is social acceptance of genetically modified animals and the regulatory policies that govern them. Additionally, establishing an international system for assessing the safety of genetically modified animals remains a significant barrier.

To facilitate the transition of genetically modified products from the laboratory to the market, it is crucial to enhance public awareness regarding their safety and ethics. Transparency and effective communication by regulators and other government agencies can help accelerate this process. Furthermore, development of well-designed regulatory policies and a scientifically grounded international system for evaluating the safety of genetically modified animals is vital for the future of animal agriculture.

## Data Availability

Not applicable.
